# A chromosome-scale genome assembly of the grape powdery mildew pathogen *Erysiphe necator* reveals its genomic architecture and previously unknown features of its biology

**DOI:** 10.1128/mbio.00645-23

**Published:** 2023-06-21

**Authors:** Alex Z. Zaccaron, Tara Neill, Jacob Corcoran, Walter F. Mahaffee, Ioannis Stergiopoulos

**Affiliations:** 1 Department of Plant Pathology, University of California Davis, Davis, California, USA; 2 USDA-ARS, Horticultural Crops Disease and Pest Management Research Unit, Corvallis, Oregon, USA; Universidade de Sao Paulo, Ribeirao Preto, Sao Paulo, Brazil

**Keywords:** Erysiphales, genome architecture, biotrophic lifestyle, transposable elements, gene duplications, copy number variation

## Abstract

*Erysiphe necator* is an obligate fungal pathogen that causes grape powdery mildew, globally the most important disease on grapevines. Previous attempts to obtain a quality genome assembly for this pathogen were hindered by its high repetitive DNA content. Here, chromatin conformation capture (Hi-C) with long-read PacBio sequencing was combined to obtain a chromosome-scale assembly and a high-quality annotation for *E. necator* isolate EnFRAME01. The resulting 81.1 Mb genome assembly is 98% complete and consists of 34 scaffolds, 11 of which represent complete chromosomes. All chromosomes contain large centromeric-like regions and lack synteny to the 11 chromosomes of the cereal PM pathogen *Blumeria gramini*s. Further analysis of their composition showed that repeats and transposable elements (TEs) occupy 62.7% of their content. TEs were almost evenly interspersed outside centromeric and telomeric regions and massively overlapped with regions of annotated genes, suggesting that they could have a significant functional impact. Abundant gene duplicates were observed as well, particularly in genes encoding candidate secreted effector proteins. Moreover, younger in age gene duplicates exhibited more relaxed selection pressure and were more likely to be located physically close in the genome than older duplicates. A total of 122 genes with copy number variations among six isolates of *E. necator* were also identified and were enriched in genes that were duplicated in EnFRAME01, indicating they may reflect an adaptive variation. Taken together, our study illuminates higher-order genomic architectural features of *E. necator* and provides a valuable resource for studying genomic structural variations in this pathogen.

**IMPORTANCE:**

Grape powdery mildew caused by the ascomycete fungus *Erysiphe necator* is economically the most important and recurrent disease in vineyards across the world. The obligate biotrophic nature of *E. necator* hinders the use of typical genetic methods to elucidate its pathogenicity and adaptation to adverse conditions, and thus comparative genomics has been a major method to study its genome biology. However, the current reference genome of *E. necator* isolate C-strain is highly fragmented with many non-coding regions left unassembled. This incompleteness prohibits in-depth comparative genomic analyses and the study of genomic structural variations (SVs) that are known to affect several aspects of microbial life, including fitness, virulence, and host adaptation. By obtaining a chromosome-scale genome assembly and a high-quality gene annotation for *E. necator*, we reveal the organization of its chromosomal content, unearth previously unknown features of its biology, and provide a reference for studying genomic SVs in this pathogen.

## INTRODUCTION

*Erysiphe necator* (Ascomycetes; Leotiomycetes, Erysiphaceae) is an obligate biotrophic fungal pathogen that causes grapevine powdery mildew (GPM), one of the most common and economically important fungal diseases in vineyards around the globe (1). The pathogen can significantly reduce grape yield and quality, and most cultivated varieties of grapevine (*Vitis vinifera*) are susceptible to it (1 - 3). As a consequence, GPM is commonly managed by fungicides, which dramatically increase the overall production costs and the risk of resistance development (4, 5).

The obligate nature of powdery mildews (PMs) prohibits the functional analysis of their genes by means of standard genetic manipulations. Instead, comparative and population genomics have been used as alternatives for studying the molecular mechanisms underlying obligate biotrophy, pathogenicity, and other aspects of the biology of these pathogens (6 - 12). To date, the genomes of at least 16 species or formae speciales of PMs have been obtained, including 3 monocotyledonous-infecting and 13 dicotyledonous-infecting species. However, the highly repetitive nature of these genomes has posed major challenges to the construction of high-quality genome assemblies based on short sequencing reads alone and has further hindered in-depth comparative genome analyses. As a result, chromosome-scale genome assemblies have, so far, only been obtained from just two monocot-infecting species of PM, namely the wheat pathogen *Blumeria graminis* f.sp. *tritici* and the triticale pathogen *B. graminis* f.sp. *triticale* (9, 13) but none from dicot-infecting PMs.

Despite challenges in obtaining high-quality genome assemblies and annotations for PM fungi, analysis of their genomic content has shown that they possess some of the largest genomes among filamentous ascomycetes, with sizes typically ranging from 120 to 180 Mb (6, 9, 14, 15). The increase is due to the extensive proliferation of transposable elements (TEs) in their genomes, which typically comprise up to 85% of their genomic content (6, 7, 9, 11). However, contrary to their enlarged genomes, PMs have a reduced number of circa (ca.) 7,000 genes (11), which are considerably smaller compared with the ca. 11,000 genes typically present in non-obligate fungal plant pathogens (16). The reduction is due to marked losses in genes encoding key enzymes in primary and secondary metabolism as well as in hydrolytic enzymes that cause damage to host cells during infection, a hallmark of their obligate biotrophic lifestyle (10, 17). PMs also lack a repeat-induced point mutation (RIP) defense mechanism against the deleterious effects caused by TE replication in their genomes (18, 19). As a consequence, their genomes experience higher rates of TE and gene duplication and retention, as these are more prone to pseudogenization in species with an active RIP mechanism. An examination of their genome architecture has further shown that PM genomes are generally deprived of large-scale compartmentalization, AT-rich isochores, and accessory chromosomes, which constitute signatures of “plastic” or “two-speed” genomes (6, 9). Instead, they adhere mostly to the “one-speed” model of genome evolution, in which gene duplication is an important mechanism of evolution and adaptation (20).

Previous efforts to sequence the genome of *E. necator* were constrained by its highly repetitive nature and the limitations of the short-read sequencing technologies used at the time. Consequently, the current reference genome of *E. necator* isolate C-strain (C-strain) is estimated to be 36.5%–48.6% complete and is assembled into 5,935 scaffolds, which forbid a rigorous analysis of its architecture (7). In this study, we present a chromosome-scale genome assembly and gene annotation for *E. necator* isolate FRAME01 (EnFRAME01). The new reference genome of *E. necator* presented herein is the first chromosome-scale assembly obtained for a dicot-infecting PM species and elucidates major aspects of their biology.

## MATERIALS AND METHODS

A detailed version of materials and methods is provided in Supplementary Results at https://doi.org/10.5281/zenodo.7738565.

### Fungal isolate, nucleic acid extraction, and sequencing

*E. necator* isolate EnFRAME01 was isolated from greenhouse-grown grapes in Corvallis, Oregon, USA, in 2018. EnFRAME01 was propagated by dusting from detached leaves (21) on *V. vinifera* L., cv. “Chardonnay” seedlings grown hydroponically in half-strength Hoagland’s solution (22). High-molecular weight DNA was obtained from conidia as in Feehan et al. (23) with modifications. PacBio library construction and sequencing were outsourced to the DNA Technologies and Expression Analysis Core Laboratory at the UC Davis Genome Center. The constructed library was sequenced using two SMRT (Single-Molecule Real Time sequencing) Cells 1M v2 on a Sequel Chemistry v2 platform (Pacific Biosciences, Menlo Park, CA, USA). Extracted DNA was also used to generate an Illumina WGS library and a Hi-C library using the Proximo Hi-C Kit (microbial) (Phase Genomics), according to the manufacturer’s instructions. Both Illumina libraries were sequenced on a NovaSeq 6000 instrument (PE150 format). To assist gene prediction, total RNA was extracted from conidia of *E. necator* isolate EnFRAME01 held at the USDA-ARS Horticultural Crops Disease and Pest Management Research Unit in Corvallis, Oregon, using Trizol reagent (ThermoFisher) according to the manufacturer’s instructions. Sample integrity analysis, cDNA library preparation, and sequencing on the Illumina NovaSeq 6000 platform using the paired-end (PE150) format were carried out at Novogene, Inc. (Sacramento, CA, USA).

### Genome assembly and annotation of repetitive DNA

PacBio reads were assembled with Canu v1.8 (24) and then used to polish the contigs with pbmm2 and Arrow from the GenomicConsensus package v2.3.3. The assembly was further polished with Pilon v1.23 (25) after mapping the Illumina reads with BWA-MEM v0.7.17 (26). Hi-C reads were mapped with BWA-MEM v0.7.17, and chromatin interaction frequencies were estimated with the 3D-DNA package (27). They were then visualized with Juicebox v1.11.08 (28), which allowed the grouping of contigs into putative chromosomes. Repetitive regions were identified with RepeatModeler v2.0.2a (29) and masked with RepeatMasker v4.1.2-p1. The repeat divergence landscape was estimated with the script parseRM.pl v5.8.2.

### Gene prediction

RNA-seq reads were mapped to the genome assembly with HISAT2 v2.2.0 (30), and transcripts were reconstructed with Stringtie v2.1.1 (31) and Trinity v2.9.1 (32). Genes were predicted with Maker v2.31.10 (33) by integrating (i) the trained *ab initio* predictors GeneMark-ES v4.57 (34), SNAP v2013-11-29 (35), and Augustus v3.2.3 (36), (ii) gene models generated with GeMoMa (37), (iii) assembled transcripts, and (iv) protein sequences from close relative species.

### Homology-based functional annotations

Conserved PFAM domains were identified with InterProScan v5.32-71.0 (38) or the NCBI CDD database (39). Carbohydrate-active enzymes (CAZymes) were predicted with dbCAN2 (40). Proteases and transporters were classified based on the top BLASTp hit (E-value <1E-10) against the MEROPS database v12.1 (41) and the TCDB database version of 2020-07-12 (42), respectively. Secreted proteins (SPs) were predicted with SignalP v5.0 (43). Membrane-bound proteins were predicted with PredGPI (44) and TMHMM v2.0 (45). Secreted proteins were classified into CSEPs based on three lines of evidence (Fig. S21): (i) EffectorP v2.0 (46); (ii) proteins shorter than 250 aa with at least 2% cysteines; and (iii) proteins with no homologs in Leotiomycetes, except Erysiphales, based on a BLASTp search (E-value <1E-3) against 93 Leotiomycetes genomes.

### Identification of core genes missing in EnFRAME01

Protein sequences from *E. necator*, *B. graminis* f.sp. *hordei*, *B. cinerea, Zymoseptoria tritici*, *Aspergillus niger*, *Neurospora crassa*, and *Saccharomyces cerevisiae* were organized into orthogroups with OrthoFinder v2.5.4 (47). Orthogroups containing proteins from all non-PM species but not from *E. necator* were considered core genes missing in *E. necator*.

### Classification and enrichment of duplicated genes

Duplicated genes were identified based on an *all-vs-all* BLASTp (E-value < 1E-5) search, with minimum identity of 40% and minimum coverage of 50%. The script *duplicate_gene_classifier* from MCScanX (48) was used to classify gene duplications into dispersed, proximal, or tandem. Enrichment of gene categories within duplicated genes was performed with hypergeometric tests using the *phyper* function within R v4.1.2. Pairwise *K_A_/K_S_* ratios were estimated with *K_A_/K_S_* _calculator v3 (49). Conserved domain enrichment was performed with the *enricher* function from the R package clusterProfiler v4.2.2 (50) within R v4.1.2 with adjusted *P*-value <0.01.

### Identification of CNVs

Whole-genome sequencing (WGS) reads of five *E. necator* isolates (7) were mapped to the genome with BWA-MEM v0.7.17 (26). PCR duplicates were marked with samblaster v0.1.24 (51) and removed with SAMtools v1.9 (52). Copy number variation (CNV) regions were identified with CNVnator (53). Genes with at least 80% overlapping with CNV regions were considered CNV genes.

### Comparative analysis of carboxylesterases

The predicted carboxylesterase (CE) HI914_00624 was queried with BLASTp against the NCBI nr database (2022, 08-13), and proteins from EnFRAME01 and the 400 most similar sequences (E-value <1E-50) were obtained. The acetylcholinesterase DmAChE from *Drosophila melanogaster* (1QO9) (54) was included as an outgroup and also used as reference to identify conserved residues. The 401 amino acid sequences were aligned with MAFFT v7.490 (55), and sites composed of more than 50% gaps were removed with trimAl v1.4 (56). The phylogenetic tree was inferred with IQ-TREE v1.6.12 (57) using the built-in ModelFinder (58) and 1,000 rapid bootstrap replicates (59). The tree was visualized and edited with iTOL (60). Quantitative PCR (qPCR) and quantitative reverse transcription PCR (RT-qPCR) were used to determine the copy number and gene expression of the *HI914_00624* gene, respectively, in six isolates of *E. necator*. qPCR reactions were run in triplicate on an Applied Biosystems QuantStudio5 qPCR machine using PerfeCTa qPCR ToughMix Low ROX (Quantabio) and the primers and probes listed in Table S26.

## RESULTS

### The genome of *E. necator* consists of 11 chromosomes with large centromeric-like regions

**Fig 1:**
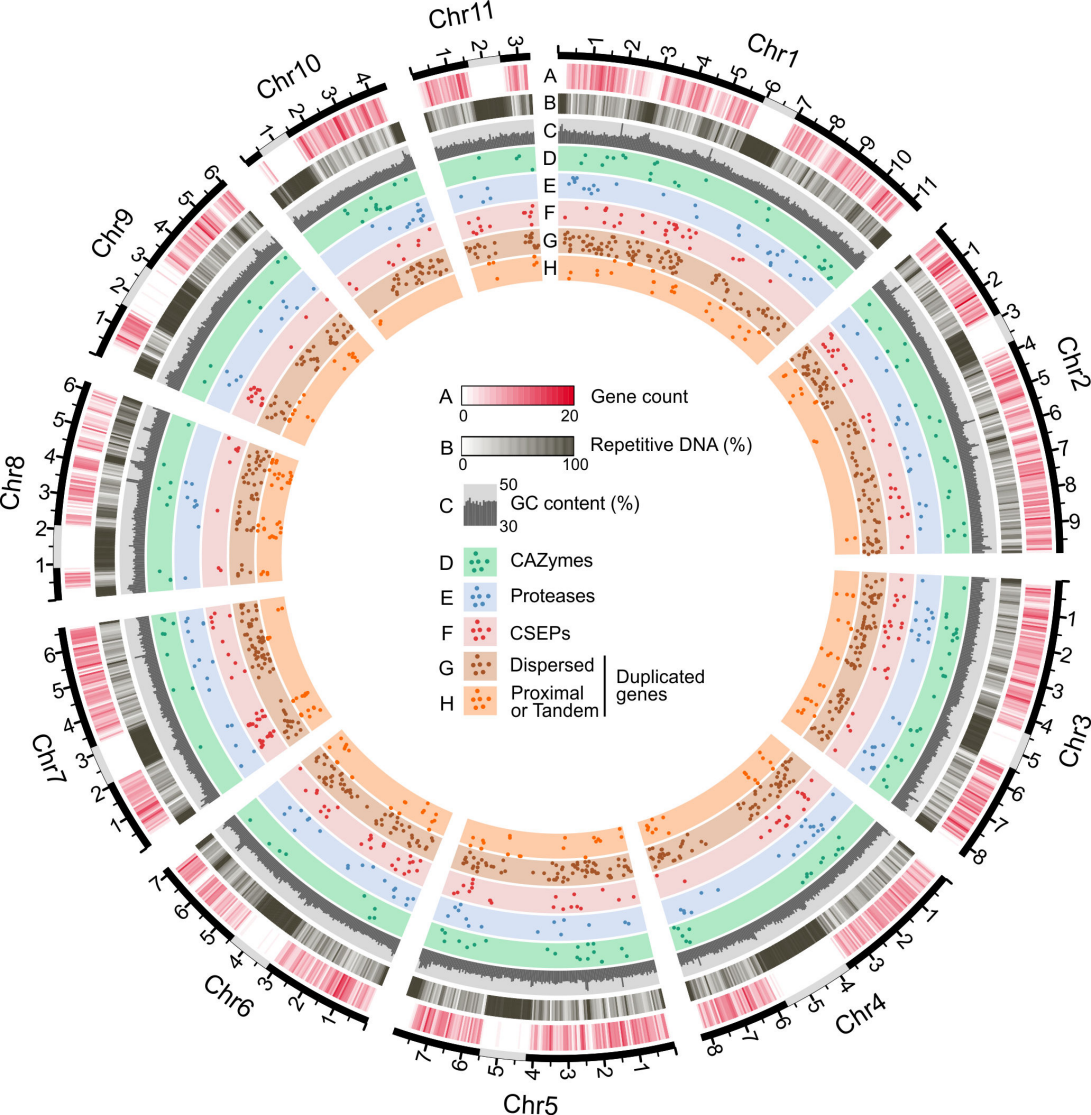
Schematic representation of the 11 chromosomes of *E. necator* isolate EnFRAME01. The Circos plot shows the assembled chromosomes as solid black lines with major tick marks representing Mb. Predicted location of centromeric regions is indicated with gray rectangles. The outermost-to-innermost tracks represent (**A**) density of protein-coding genes, (**B**) repetitive DNA content, (**C**) GC content from 30% to 50%, (**D**) location of genes encoding carbohydrate-active enzymes, (**E**) location of genes encoding proteases, (**F**) location of genes encoding CSEPs, (**G**) location of dispersed gene duplicates (i.e., gene copies located in different chromosomes or separated by more than 10 genes), and (**H**) location of proximal or tandem gene duplicates (i.e., gene copies located less than 10 genes apart or next to each other). Gene locations are represented by bullet points on the perpendicular axis. Gene count, repetitive DNA, and GC content were determined using a sliding window of 50 kb. The figure shows that the chromosomes of EnFRAME01 contain long centromeric regions, which are abundant in repeats and nearly devoid of protein-coding genes.

The genome of EnFRAME01 was assembled using a combination of PacBio reads and Hi-C data into 34 scaffolds, totaling 81.1 Mb in size. This is a considerable improvement over the previous reference genome of C-strain that was fragmented into 5,935 scaffolds (Table S1 and Supplementary Results). Of the 34 assembled scaffolds, 11 embodied distinct chromosomes (Chr1-to-Chr11) (Fig. 1; Fig. S1; Table 1), 22 were unplaced scaffolds, and 1 scaffold represented the complete mitochondrial genome (Fig. S2). The size of the 11 chromosomes ranged from 11.3 Mb (Chr1) to 3.3 Mb (Chr11), and all were putatively assembled telomere-to-telomere, containing only five collapsed regions (Fig. S3). All chromosomes had 22–31 copies of the canonical telomeric repeat 5′-TTAGGG-3′ at their ends and predicted centromeric regions with high inter-chromosomal Hi-C contact frequency (Fig. S1A), as previously observed in other fungi (61, 62). However, in contrast to other ascomycetes (61, 63 - 66), the predicted centromeres of *E. necator* were large segments that accounted for 15.8% of the genome (Table 1 and Table S2). Centromeric regions of similar sizes have been reported before for the wheat PM *B. graminis* f.sp. *tritici* (9), but whole-genome alignment showed that the predicted centromeric regions of *E. necator* are poorly conserved in *B. graminis* f.sp. *tritici*. Moreover, although both PM species have 11 chromosomes, they exhibited an overall low synteny as no one-to-one chromosome match was observed between them (Fig. S4). These results indicate poor conservation of centromeric regions and extensive inter-chromosomal rearrangements between *E. necator* and *B. graminis* f.sp. *tritici*.

**TABLE 1:** Size and content of the 11 chromosomes of *Erysiphe necator* isolate EnFRAME01

Chromosome	Size (Mb)	GC (%)	Centromere size (Mb)	Predicted genes	Genes per Mb	Median intergenic size (bp)	Repeats (%)
Chr1	11.30	39.8	1.00	1,000	88	4,333	61.7
Chr2	9.87	39.6	0.85	1,060	107	3,391	55.6
Chr3	8.28	39.5	1.05	840	101	3,502	58.4
Chr4	8.27	39.6	2.00	684	83	4,000	64.4
Chr5	7.98	39.8	1.30	758	95	3,286	61.8
Chr6	7.15	39.8	1.25	608	85	4,402	64.9
Chr7	6.66	39.4	1.12	641	96	3,639	60.5
Chr8	6.21	40.2	1.20	393	63	5,218	70.0
Chr9	6.07	39.9	1.25	474	78	3,249	69.4
Chr10	4.49	39.5	0.90	387	86	3,536	63.6
Chr11	3.34	39.4	0.90	261	78	3,666	68.3

### A reduced gene complement underlies the obligate biotrophic lifestyle of *E. necator*

A total of 7,146 protein-coding genes were predicted in the genome of EnFRAME01, with a BUSCO completeness of 98.2%. This gene number is comparable with the 6,046–8,470 genes reported in other PM fungi (6, 8, 9, 67), and a notable improvement over the gene annotation of C-strain, for which 6,484 genes were predicted with an estimated completeness of 90.1% (Table S3). Functional gene annotations showed that the genome of EnFRAME01 contained 174 proteases (Table S4), 8 key enzymes for secondary metabolism (Table S5), 11 cytochrome P450s (Fig. S5; Table S6), 1,238 putative transporters (Fig. S6; Table S7), 160 CAZymes (Table S8), and 527 SPs (Table S9), of which 234 were candidate secreted effector proteins (CSEPs) (Table S10) (Supplementary Results). The number of genes in these functional categories is low compared with other plant pathogenic ascomycete fungi (8, 68) but similar to PMs (8, 15). Consistent also with an obligate biotrophic lifestyle, 181 core genes were identified that are typically present in *S. cerevisiae* and non-obligate biotrophic fungi but were missing in EnFRAME01 (Table S11). Included in these genes were 95 of the 99 so-called “missing ascomycete pathogen core genes,” generally reported as absent in PMs (10) (Table S12). Based on KEGG orthology (KO) identifiers (69), the 181 genes are predicted to partake in 47 conserved pathways (Table S13), of which 23 were significantly enriched in genes missing in EnFRAME01 (Supplementary Results). The two pathways most affected by gene losses were thiamine and sulfur metabolism, in accordance to other obligate biotrophic fungi (10, 17, 68). Absence of a sterol O-acyltransferase (EC:2.3.1.26) gene, and of the *ERG5* (C-22 sterol desaturase; EC:1.14.19.41) and *ERG4* (EC:1.3.1.71) genes whose products catalyze the last two steps of ergosterol biosynthesis in yeast (70), was also observed. Collectively, these results indicate that the obligate lifestyle of *E. necator* is driven by losses in genes involved in several biochemical pathways, in accordance to what has been observed in other PMs and obligate biotrophs (10, 17).

### *E. necator* harbors a reduced arsenal of CSEPs

The small number of 234 CSEP-encoding genes identified in the genome of EnFRAME01 is in line with reports from dicot-infecting PMs but in contrast to monocot-infecting PMs such as different *B. graminis* formae speciales (6, 9). Of the 234 CSEPs, 49 (20.9%) were species specific, and 185 (79.1%) had homologs in PMs (*n* = 183) and/or non-PM fungi (*n* = 86) (Fig. S7). Moreover, 86 (36.7%) contained the Y/*F*/WxC sequence motif that is typically found in CSEPs of *B. graminis* and other PMs (Fig. S8 and Table S10). PM fungi are also known to harbor many ribonuclease-like effectors that belong to a large family of catalytically inactive RNases, known as RALPHs (RNase-like proteins associated with haustoria) (71 - 73). A genome-wide search in EnFRAME01 identified 38 genes encoding RALPH-like proteins, 24 of which could also be classified as CSEPs (Table S14). A phylogenetic analysis grouped the 38 RALPH-like proteins into two major clades, whose members differed in average protein size and the location in the genome of their encoding genes (Fig. S9). An Egh16-like virulence factor domain (PF11327) was also commonly found in the *E. necator* CSEPs. CSEPs with an Egh16 domain are members of a multigene family in fungi (74, 75) and often play a role during the early stages of host infection. A total of 11 genes encoding Egh16-like proteins that could be further clustered into two clades (Fig. S10) were identified in EnFRAME01, but only four of these were classified as CSEPs (Table S15). Finally, an analysis of the localization of the 234 CSEP-encoding genes of *E. necator* on the 11 chromosomes of the fungus showed there was no enrichment of genes encoding CSEPs in subtelomeric regions, as has been observed in other fungi (76, 77) (Fig. 1).

### TE bursts have drastically shaped the genome of *E. necator*

**Fig 2:**
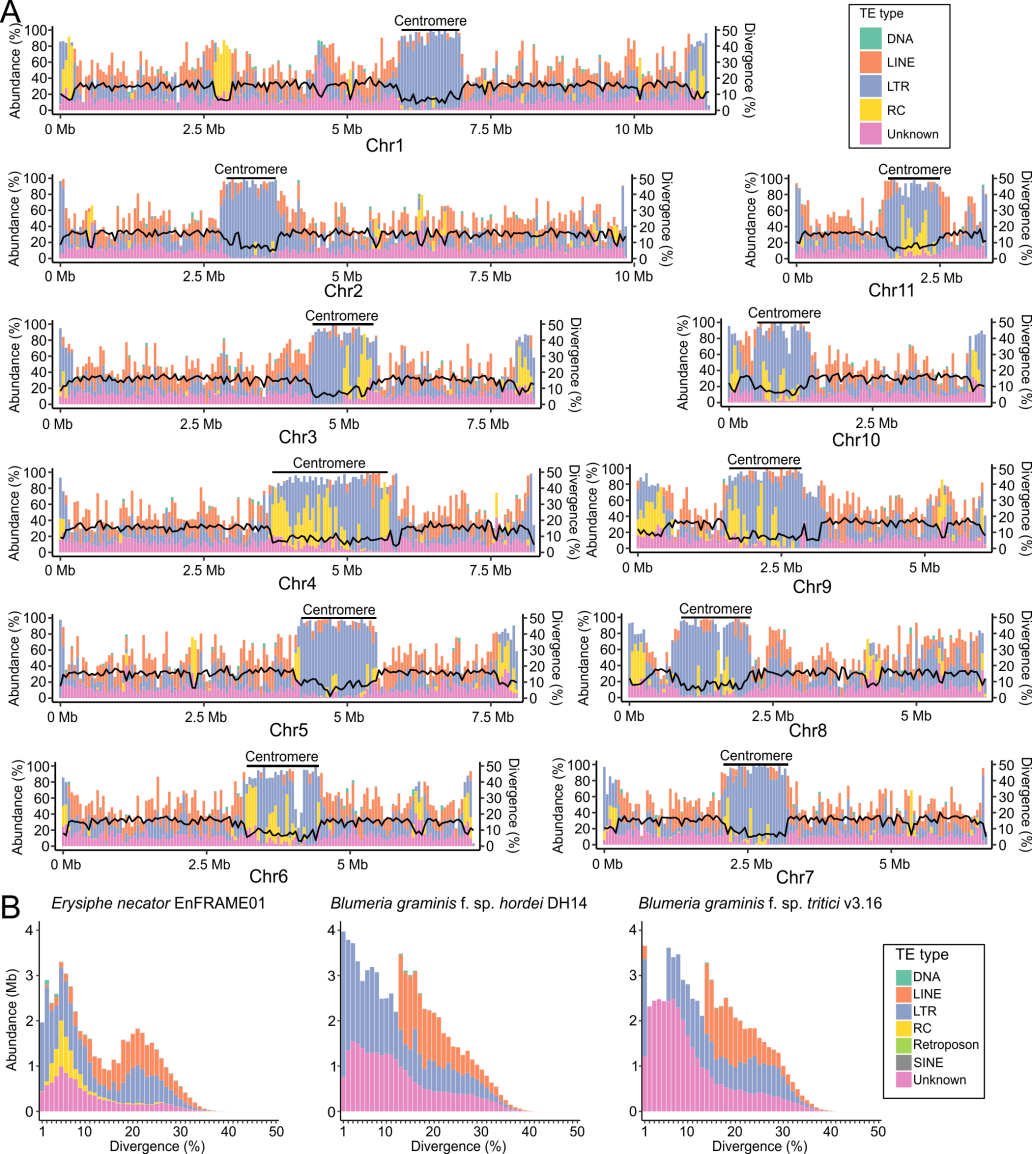
The transposable element composition of *E. necator* differs from that of the cereal powdery mildew pathogens *B. graminis* f.sp. *hordei* and *B. graminis* f.sp. *tritici*. (**A**) Distribution of TEs in the 11 chromosomes of *E. necator* isolate EnFRAME01. The figure shows the abundance of the different TE classes, represented as stacked bar plots along the chromosomes. Overall divergence of TE families is indicated by solid black lines along the chromosomes. Predicted centromeric regions are indicated as well. The figure shows high abundance of repeats near chromosome ends and at centromeres. Predicted centromeric regions are enriched mainly in long terminal repeat retrotransposons with overall low sequence divergence compared with the rest of the genome. Rolling-circle elements are also abundantly found in centromeres and have an overall low sequence divergence. TE abundance and divergence were calculated using a sliding window of 50 kb. (**B**) Repetitive DNA landscape represented as bar plots showing the number of bases covered by predicted TEs from different (sub)classes. The predicted divergence of the TEs is shown on the *x*-axis. The figure shows a bimodal repeat divergence landscape with peaks for *E. necator* at approximately 5% and 21% divergence. The two peaks differ in their composition, with the peak at 5% divergence being dominated by LTRs, RCs, and unknown elements, and the peak at 21% divergence being dominated by LTR and long interspersed nuclear elements (LINE). The landscape of TE divergence of the cereal PM pathogens also follows a bimodal distribution, but it is less pronounced as compared with *E. necator,* and the peaks are void of RCs.

The genome of EnFRAME01 is highly repetitive, with repeats accounting for 62.7% (50.8 Mb) of its DNA content. Class I retrotransposons, such as long terminal repeat (LTR) retrotransposons (26.5%, 21.5 Mb) and non-LTR retrotransposons (16.8%, 13.6 Mb), were more abundant than class II DNA transposons (6.3%, 5.1 Mb) and unclassified interspersed repeats (13.1%, 10.6 Mb) (Table S16). This is consistent with most fungi (78, 79) but in contrast to cereal PMs*,* whose genomes are mainly dominated by non-LTR retrotransposons (6, 9). TEs were fairly evenly dispersed outside centromeric and subtelomeric regions, which generally contained smaller amounts of non-LTR elements and exhibited an overall lower TE divergence. A similar pattern was also observed in genomic islands rich in rolling-circle (RC) elements (Fig. 2A). Collectively, these observations indicate that younger TEs accumulated preferentially in centromeric and subtelomeric regions and that RC elements are younger than other TEs (Fig. 2A). Interestingly, an examination of the nucleotide divergence among TE copies revealed a bimodal distribution with two peaks of contrasting TE composition. This suggests the presence of two TE burst events in the evolutionary history of *E. necator* that involved different TE classes (Fig. 2B and Supplementary Results). A similar pattern was also observed in the genomes of different *B. graminis* formae speciales, although the bimodal peaks were less pronounced and lacked RC elements (Fig. 2B; Table S16). In addition, highly divergent TEs were in all genomes enriched in non-LTR rather than LTR elements, whereas the opposite was observed for TEs with low divergence. By using the *E. necator* repeat library to mask the genomes of the cereal PMs, and vice versa, nearly all low-divergence TEs were left unmasked (Fig. S11). These observations suggest that *E. necator* and *B. graminis* underwent a similar burst of non-LTR TEs, possibly prior to their divergence, followed by clade-specific proliferation of LTR-retrotransposons and, in the case of *E. necator*, of RC elements as well.

### The genome of *E. necator* exhibits small-scale compartmentalization

**Fig 3:**
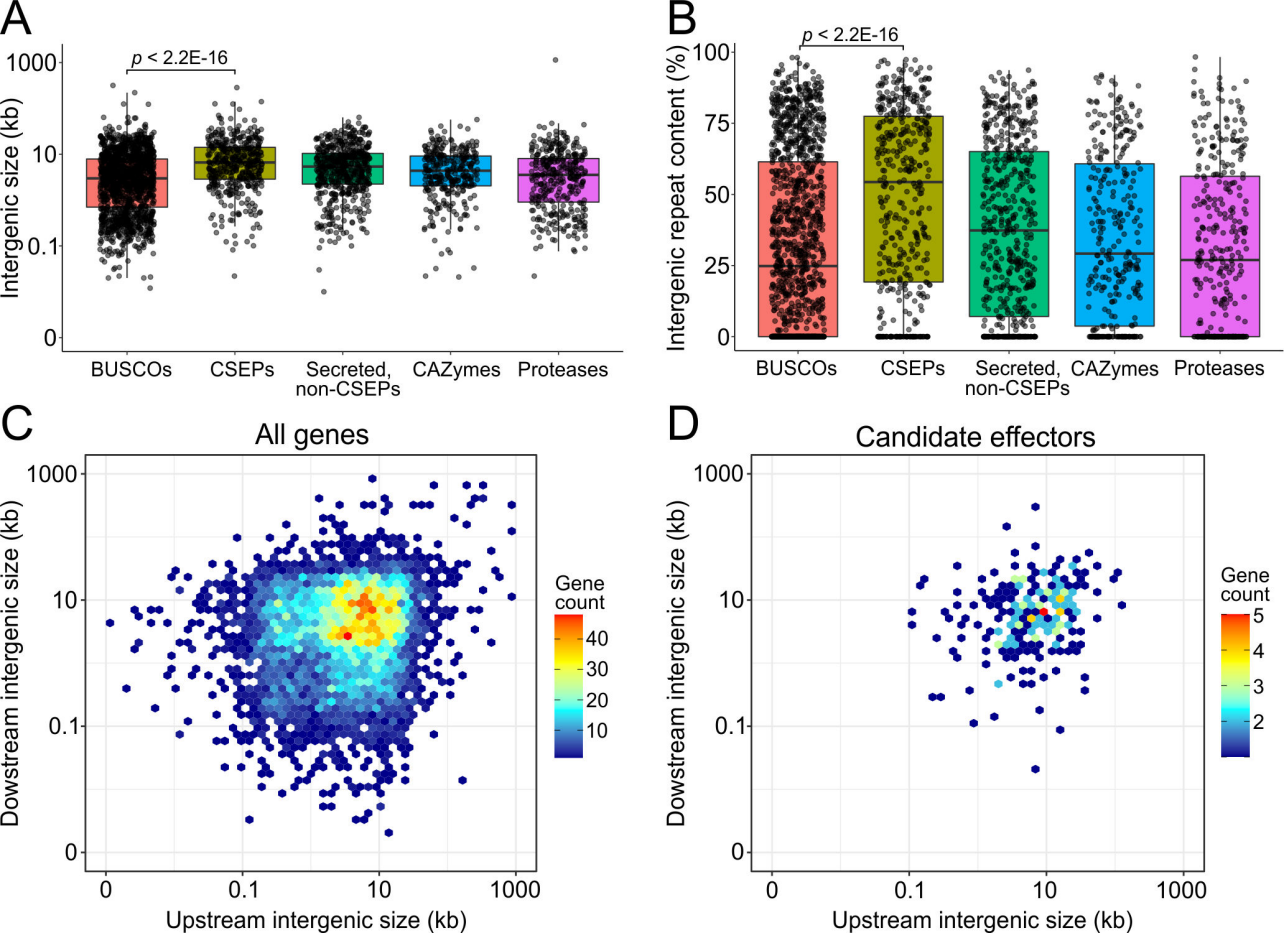
The genome of *E. necator* isolate EnFRAME01 exhibits small-scale compartmentalization of genes encoding candidate secreted effector proteins in repeat-rich genomic regions. (**A and B**) Boxplots showing the size distribution and repetitive DNA content of upstream and downstream intergenic regions flaking BUSCO genes, genes encoding CSEPs, genes encoding secreted proteins not classified as CSEPs, genes encoding carbohydrate-active enzymes, and genes encoding proteases. The figure shows that intergenic regions of CSEP-encoding genes typically have higher repetitive DNA content compared with genes in the other categories. The *P*-values shown in panels A and B were obtained with the Wilcoxon rank sum test. (**C and D**) Heatmaps of the number of protein coding genes (panel C) and CSEPs (panel D) with certain sizes of upstream (*y*-axis) and downstream (*x*-axis) intergenic regions. The figure shows that the genome of EnFRAME01 does not exhibit large-scale compartmentalization of CSEP-encoding genes in gene-sparse regions.

An examination of the distribution of repeats and of protein coding genes on the 11 chromosomes of EnFRAME01 revealed large differences in gene density among the chromosomes and an inverse correlation between density of protein coding genes and repetitive DNA content (Fig. 1; Fig. S12 and Supplementary Results). An assessment of whether certain gene categories were associated with specific TE superfamilies showed no major differences in TE content within the flanking regions of genes encoding CAZymes, proteases, CSEPs, and non-CSEP secreted proteins (Fig. S13). However, the intergenic regions of CSEP genes were significantly longer, richer in repetitive DNA, and had a different TE composition as compared with other functional gene categories (Fig. 3A and B; Table S17). Collectively, these observations indicate the absence of large-scale compartmentalization in gene-dense and gene-sparse regions in the genome of *E. necator* (Fig. 3C and D), consistent with the “one-speed” genome hypothesis suggested for PM species (6, 20). Instead, small-scale compartmentalization of CSEP-encoding genes was seen, which was preferentially located in somewhat gene-sparse and repeat-rich regions, as commonly reported in other fungal pathogens (80, 81).

### Gene duplication asymmetrically affects different functional gene categories in *E. necator* and their genomic organization

**Fig 4:**
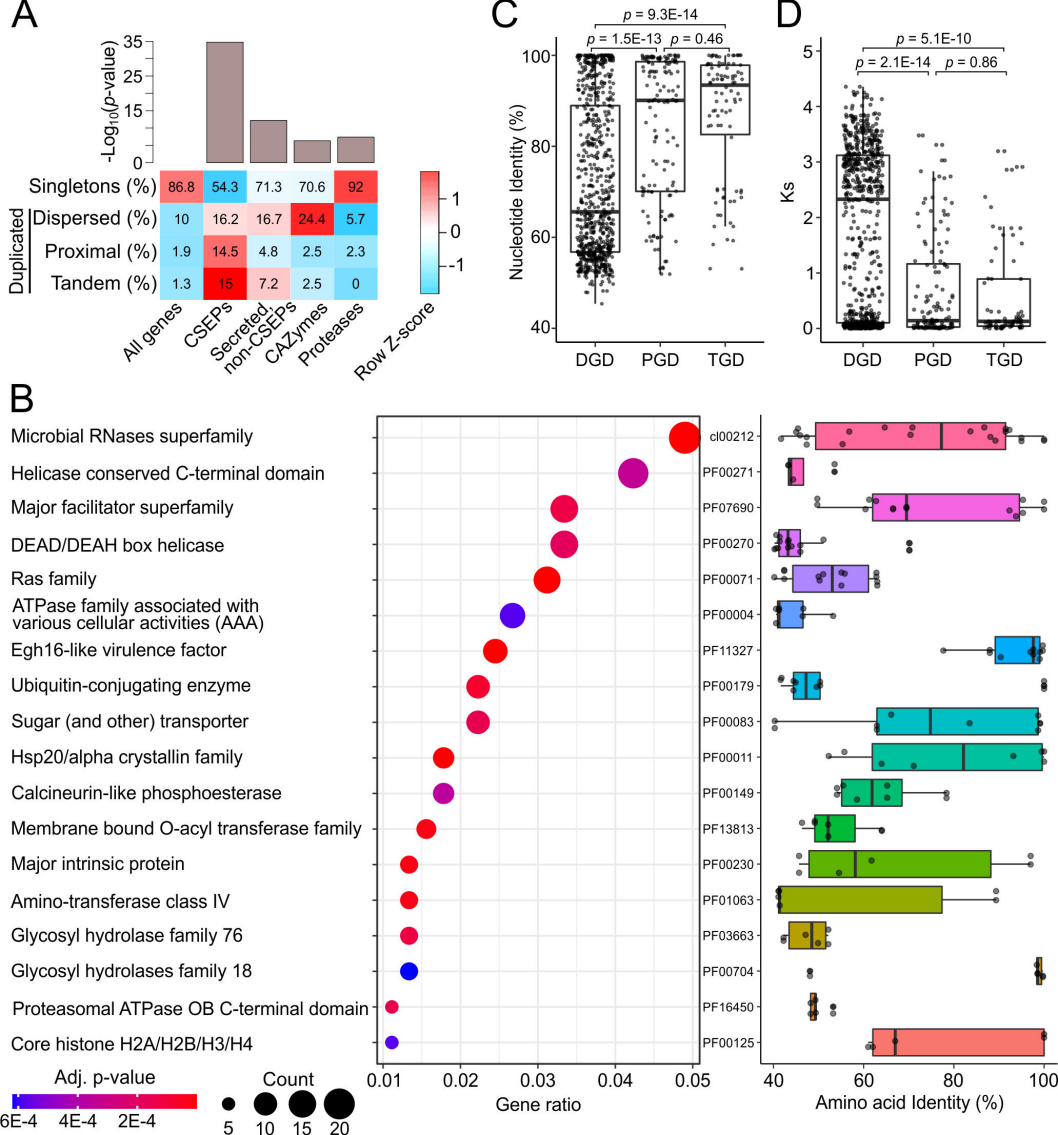
Landscape of gene duplications in the genome of *E. necator* isolate EnFRAME01. (**A**) Heatmap showing the percentage of genes in different functional categories that are singletons, dispersed duplications, proximal duplications, and tandem duplications. The bar chart shows *P*-values for enrichment of duplicated genes based on hypergeometric tests. The figure shows that ~13% of the genes are duplicated and that the percentages of duplicated genes encoding candidate secreted effector proteins are significantly higher than genes encoding secreted proteins not classified as CSEPs, carbohydrate-active enzymes, and proteases. (**B**) The dot plot shows conserved domains significantly enriched within duplicated genes. The size of the dots corresponds to the number of duplicated genes containing the respective domain. The *x-*axis shows the proportion of the duplicated genes containing the respective domain that contributes to all duplicated genes containing a conserved domain. Dots are color coded based on enrichment *P*-values adjusted using the Benjamini–Hochberg method. Distributions of pairwise identity values of duplicated copies are shown on the right-hand side based on top BLASTp hit. The *P*-values shown in (**C**) and (**D**) were obtained with the Wilcoxon rank sum test. (**C**) Boxplots showing the distribution of pairwise nucleotide identity values of dispersed gene duplicates, proximal gene duplicates, and tandem gene duplicates. Each point represents a duplicated gene with the percent identity of its top BLASTn hit shown in the *y*-axis. The figure shows that copies of DGDs share significantly less nucleotide identity (median = 65.6%) than copies of PGDs (median = 90.1%) and TGDs (median = 93.4%). (**D**) Boxplots showing the distribution of *K_A_/K_S_* values for DGDs, PGDs, and TGDs. Each point represents a duplicated gene with the *K_A_/K_S_* value of its top BLASTp hit shown in the *y*-axis. The figure shows that copies of PGDs and TGDs share higher conservation of K*_A_*/K*_S_* values than copies of DGDs.

A self-BLASTp search revealed a total of 941 genes (13.1%) duplicated in the genome of EnFRAME01, with CSEP-encoding genes experiencing significantly (*P*-value = 1.8E-35) higher rates of gene duplications, as compared with genes in other functional categories (Fig. 4A; Table S18; Supplementary Results). A conserved domain enrichment analysis further identified 30 domains that were significantly enriched among duplicated genes (adjusted *P*-value <0.01), with the two most significantly enriched being the microbial ribonuclease (cl00212) and the Egh16-like virulence factor (PF11327) domains that are associated with CSEPs as well (Fig. 4B; Table S19). When considering the arrangement of the 941 gene duplicates in the genome of *E. necator*, the majority were dispersed gene duplicates (DGDs; *n* = 712; 75.6%), as opposed to being proximal gene duplicates (PGDs; *n* = 139; 14.8%) or tandem gene duplications (TGDs; *n* = 90; 9.5%) (Fig. 4A). However, genes encoding CSEPs significantly deviated from this pattern as they exhibited almost equal frequencies of dispersed (*n* = 38; 35.5%), proximal (*n* = 34; 31.8%), and tandem (*n* = 35; 32.7%) duplications (Fig. 4A). Indeed, several of the multicopy CSEP-encoding genes, including the RALPH-like and Egh16-like CSEPs, were found to be tandemly arranged in clusters (Fig. S14), suggesting that CSEPs families expand by frequent local duplications in *E. necator*. A prominent example of this trend was the discovery of a 350-kb region on Chr1 that harbored 20 copies of a CSEP-encoding gene (i.e., *HI914_00480*), which were tandemly arranged in the same orientation on the same DNA strand (Fig. S15) and with 15 consecutive copies encoding identical proteins. Our analyses also suggested that local gene duplicates (i.e., PGDs and TGDs) are more conserved and thus more likely to contribute to genetic redundancy than DGDs, which due to their higher divergence, are likely to contribute more to functional diversification (Fig. 4C). Similarly, when examining the rate of synonymous (*K_S_*) and non-synonymous substitutions (*K_A_*), local gene duplicates exhibited overall lower *K_S_* and higher *K_A_/K_S_* values as compared with DGDs (Fig. 4D; Fig. S16). This indicated that PGDs and TGDs were likely more recent duplicates and were under more relaxed selection pressure as compared with DGDs. Collectively, the above results indicate that gene duplication is a driver of genome evolution in *E. necator* that has differentially affected different gene categories, thereby leading to differences in their mode of evolution and organization of their paralogs in the genome.

### Duplicated genes in EnFRAME01 frequently vary in copy number among *E. necator* isolates

The WGS data of five *E. necator* isolates (7) were used to identify genomic regions in EnFRAME01 with CNV (i.e., deleted or duplicated). A total of 1,760 distinct CNV regions were identified, of which 1,589 (90.3% with an average size of 2.9 kb) were deletions, and only 171 (9.7% with an average size of 5.6 kb) were duplications (Fig. S17A; Table S20). CNV regions were dispersed throughout the 11 chromosomes of EnFRAME01 (Fig. S18) and were more frequently located near chromosome ends rather than gene-rich and repeat-rich regions (Fig. S17B). However, despite the lack of enrichment of CNV regions in repeat-rich regions, 80.5% (*n* = 1,279) of the deleted and 70.1% (*n* = 120) of the duplicated regions overlapped with predicted TEs (Fig. S17C). Moreover, 122 of the CNV regions overlapped with protein-coding genes and could, therefore, be considered as CNV genes (Supplementary Results). Of these, 53 genes were duplicated (average of 0.3 duplicated gene per all duplicated regions), and 69 genes were deleted (average of 1.5E-5 deleted gene per all deleted regions) among the *E. necator* isolates (Table S21), indicating that genes with CNV were most likely to be affected by duplications rather than deletions. Most CNV regions also typically affected single genes rather than groups of genes, and no significant over- or under-representation of CSEPs, CAZymes, and proteases was observed among CNV genes. Instead, genes with CNV were significantly (*P*-value = 1.7E-27) enriched with the 941 genes predicted to be duplicated in EnFRAME01 (Table S22). A notable example is the CSEP-encoding gene *HI914_00480*, which is present in 20 copies in EnFRAME01 and 8–12 copies in other strains (Table S23). This indicated that rates of gain, retention, and loss of duplicated genes were asymmetric among different isolates of *E. necator*.

### *E. necator* exhibits extensive CNV of a novel and PM-specific CE

**Fig 5:**
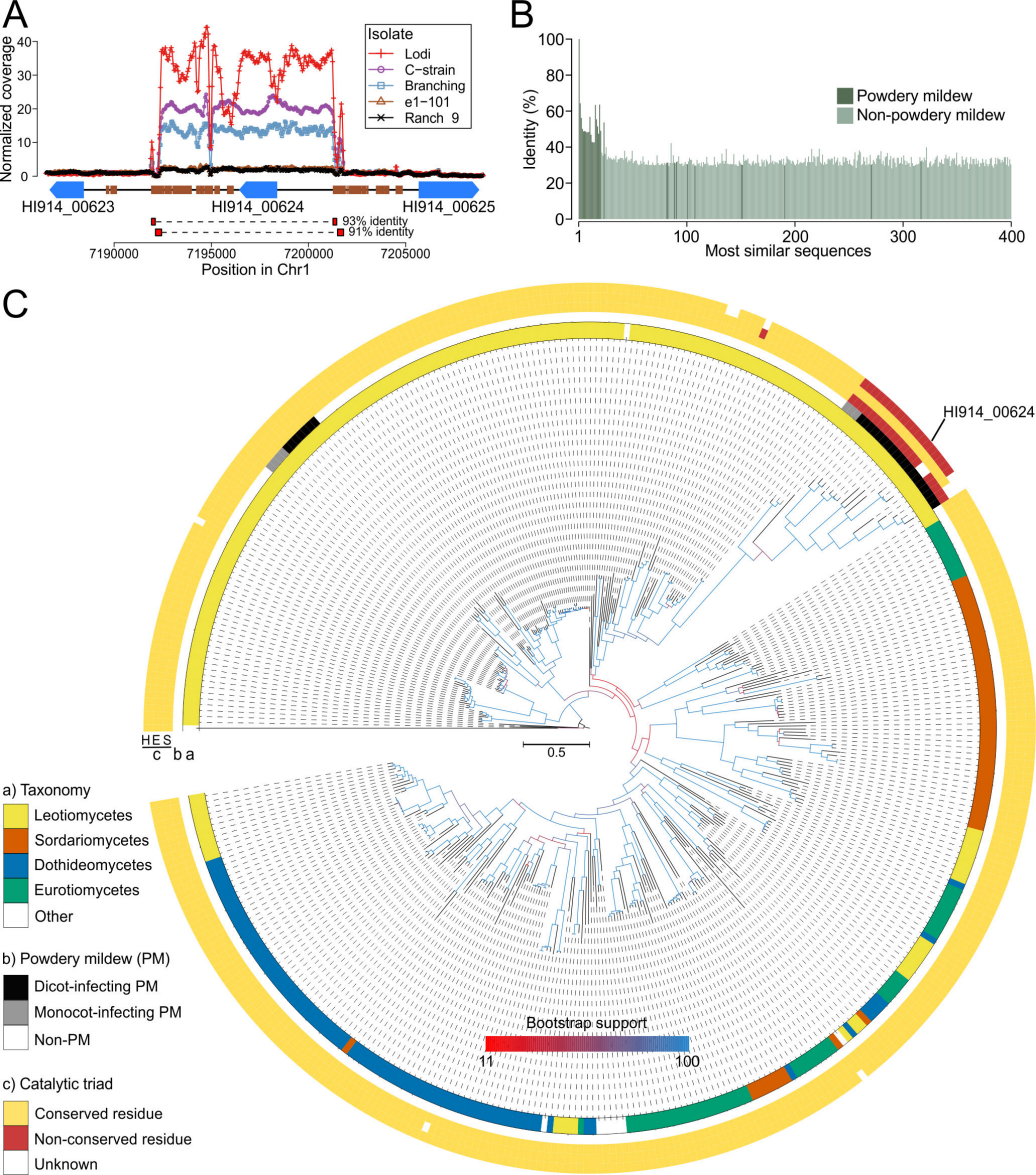
*E. necator* shows extensive copy number variation of a putative secreted carboxylesterase that is poorly conserved in non-powdery mildew fungi. (**A**) Region of chromosome 1 (Chr1) in the genome of *E. necator* isolate EnFRAME01 containing the gene *HI914_00624* encoding a putative secreted CE. Genes are represented as blue arrows and repetitive DNA as small brown rectangles. Lines above the genes indicate estimated copy numbers of the region in five different isolates. The figure shows that isolates Lodi, C-strain, and Branching have more than 10 predicted copies of *HI914_00624*. The duplicated segment is flanked by short direct repeats of more than 90% identity. The figure also shows that genes flanking *HI914_00624* are not duplicated in the isolates analyzed. (**B**) Percent identity values of most similar sequences to the HI914_00624 protein sequence based on BLASTp searches. The figure shows that nearly all sequences from non-PM species have less than 40% amino acid identity. (**C**) Maximum likelihood phylogenetic tree of HI914_00624 and its most similar protein sequences from GenBank (2022, 08-13) and EnFRAME01. The protein sequence of the acetylcholinesterase DmAChE from *D. melanogaster* (54) was included as an outgroup. Tree branches are color coded based on their support of 1,000 bootstrap replicates. The tree was rooted at DmAChE. Track (**A**) shows the distribution of taxonomy classes of the sequences. Track (**B**) indicates sequences from monocot-infecting and dicot-infecting PMs. Track (**C**) shows the conservation of the Ser, Asp/Glu, and His residues that comprise the catalytic triad conserved in CEs. The figure shows that homologs of HI914_00624 are conserved in other PMs but poorly conserved in non-PM fungal species. The figure also shows that homologs of HI914_00624 in PMs lack the Ser and His residues of the catalytic triad, which are largely conserved in predicted CEs from other fungal species.

An inspection of the 122 genes with CNVs among the isolates of *E. necator* showed that gene *HI914_00624,* encoding a predicted secreted CE, exhibited the most dynamic changes in copy numbers, ranging from 1 in isolate EnFRAME01 to 31 in isolate Lodi (Supplementary Results). Moreover, RT-qPCR assays showed that the relative expression of this gene in six 2-week-old isolates of *E. necator* was strongly correlated (Pearson’s linear correlation coefficient *r^2^* = 0.998, *P* < 0.001) to its copy number (Fig S19 and Table S24), indicating that increases in copy number resulted in a gene dosage effect. In all isolates, the duplication affected the same 9.5 kb fragment that contained only the *HI914_00624* gene and was flanked by short direct repeats (Fig. 5A). A blast search within the NCBI nr database indicated that homologs of HI914_00624 are abundantly present both within PM and non-PM fungal species (Fig. 5B). However, a phylogenetic tree constructed using the top 400 best BLASTp hits, representing at least 195 distinct fungal species, showed that HI914_00624 belonged to a distinct clade that included 22 CEs, all from PM species (Fig. 5C). Moreover, a multiple sequence alignment showed that the catalytic triad Ser-Asp/Glu-His, that is indispensable to the function of CEs (82, 83), is poorly conserved in these 22 PM-specific CEs (Fig. S20; Table S25). This suggests that HI914_00624 is a member of new clade of potentially non-catalytically active CEs or CEs with a modified enzymatic activity (84).

## DISCUSSION

In this study, we used deep WGS sequencing to obtain a chromosome-scale genome for the grape PM *E. necator*, the first Erysiphales genome for a dicot-infecting PM. The availability of a high-quality assembly enabled us to uncover prominent genomic and biological features of *E. necator* that were previously left unexplored due to its fragmented assembly. This highlights the importance of obtaining high-quality chromosome-level assemblies and further sets a solid basis for a detailed structural genomics studies in this pathogen.

The 81.1 Mb genome of *E. necator* is organized into 11 chromosomes, which are broadly characterized by the presence of large centromeric-like regions rich in repetitive DNA, a high content of retrotransposons and unclassified repeats that are mostly evenly dispersed outside their centromeric and telomeric regions, and the lack of compartmentalization in repeat-rich/gene-sparse regions, in agreement with the “one-speed genome” model of evolution. Moreover, *E. necator* had a reduced complement of genes encoding lytic enzymes (e.g., CAZymes and proteases) and those involved in carbohydrate metabolism, amino acid and purine metabolism, thiamine biosynthesis, and assimilation of inorganic nitrogen and sulfur. Loss of genes affecting these pathways is a characteristic feature of obligate biotrophs (8, 17, 68, 85, 86) and potentially a strategy used by such pathogens for conserving their resources, if the end products of the impaired pathways are available through leaky metabolic processes in the host (17, 87 - 89). This model of reductive genome evolution in which organisms abolish genes needed to synthesize metabolites that can be obtained directly through the host environment is frequently observed in nature, including obligate biotrophic fungi (68, 87, 88, 90).

The search for genes that are missing in *E. necator* but are commonly present in other ascomycete fungi also revealed that the fungus lacks *ERG5* and *ERG4*, whose products catalyze the two last steps, respectively, of ergosterol biosynthesis in fungi (70). This corroborates previous reports that ergosterol is essentially absent in this fungus as well as in PMs, in general (91, 92). The deletion of *ERG5* and/or *ERG4* is typically not lethal to fungi but leads to an altered ergosterol biosynthesis that may affect their physiology, increase their sensitivity to multiple chemicals, and generally decrease their fitness under stress conditions (93 - 97). For instance, both genes are required for the conidiation of *A. fumigatus* (98, 99), *ERG4* is crucial for vegetative differentiation and virulence in *Fusarium graminearum* (100), and deletion of *ERG4* increased the production of extracellular pigments in *Monascus purpureus* (101). Likewise, the deletion of *ERG5* increased the susceptibility of *Candida albicans, N. crassa,* and *F. verticillioides* to azole antifungals (102, 103). Loss of *ERG4* and *ERG5* is unlikely to have a fitness effect on *E. necator* or impact major features of its physiology, and it is thus intriguing to speculate possible biological explanations for the loss of ergosterol biosynthesis. As ergosterol is an inducer of innate immunity in plants (104), its absence may help *E. necator* avoid sterol-induced immunity in grapes. This might be possible as the activation in *V. vinifera* of the type I lipid transfer protein VvLTP1 by ergosterol treatment (105, 106) leads to the induction of the stilbene synthase gene *Vst1* (105). *Vst1*, in turn, regulates the biosynthesis of the phytoalexin resveratrol that enhances resistance against *Botrytis cinerea* (107) and *E. necator* (108).

The overall genomic characteristics of *E. necator* conform to those reported for other PM pathogens, including different formae speciales of the cereal PM pathogen *B. graminis* (8, 10, 14). However, the 11 chromosomes of *E. necator* are not syntenic to the 11 chromosomes of *B. graminis*, indicating rapid diversification of their genomic architecture following speciation. Moreover, the genome of *E. necator* has a different TE complement compared with *B. graminis*, as it contains mostly LTR retrotransposons rather than the non-LTR retrotransposons. TEs are a major force of evolution and adaptation to stressful environments, as their bursts and mobilization provoke chromosomal reorganization and phylogenetic divergence (109, 110). Our analysis showed that both species have experienced not one, as previously reported (6), but at least two bursts of TEs in their evolutionary history. The first burst possibly preceded their divergence and involved mostly non-LTR retrotransposons, and the second burst likely took place after their speciation and involved LTR-retrotransposons but also RCs (i.e., Helitrons) in *E. necator*. Such differences in TE bursts in *E. necator* and *B. graminis* are likely to have restructured their genomes, accelerated their speciation, and influenced their adaptation on different hosts by, among others, affecting virulence-associated genes such as CSEPs. Indeed, despite the lack of large-scale compartmentalization in *E. necator*, its CSEP-encoding genes exhibited significantly higher duplication rates as compared with other functional gene categories and were embedded in larger intergenic regions that were richer in TEs. The increase in duplication rates could have been prompted by the presence of TEs, as the repetitive nature of transposons provides a substrate for non-allelic homologous recombination that would typically generate tandemly arranged gene copies in their flanking regions (111 - 113). TEs have also been hypothesized to mediate the duplication and proliferation of CSEPs in *B. graminis* (9, 73), as CSEP-encoding genes in this species are frequently duplicated and present in tandem in physical proximity to similar repetitive DNA (9, 73). Thus, next to promoting chromosomal reorganization, TEs seem to have had a major role in shaping the evolution of *E. necator* and *B. graminis* as plant pathogens by providing a favorable environment for CSEP duplication.

The inflation of the *E. necator* genome by TEs was further accompanied by high rates of gene duplication, which likely contributed further to its genomic plasticity and genetic diversity. Gene duplication is a major force of evolution as it provides material for functional, regulatory, and transcriptional divergence through the generation of new genes and their subsequent neo-, sub-, or hypo-functionalization (114, 115). A variety of mechanisms can trigger gene duplications, with different mechanisms creating suites of duplicated genes in different configurations within a genome, which in turn contribute differentially to functional innovation and redundancy (116, 117). Our analysis indicated that in *E. necator,* genes from different functional categories exhibited different rates and modes of duplications and that different modes of gene duplication were under different strengths of selection pressure. These features were again more prominent with CSEP-encoding genes, as CSEP gene duplicates were more likely to be in close (i.e., tandem or proximal) physical location in the genome of EnFRAME01 than the copies of other gene classes and had on average higher *K_A_/K_S_* values, indicating that duplicated CSEP genes are potentially subject to higher rates of evolution. This is consistent with the role of effectors on host adaptation and overcoming of the host immune system and indicates an ongoing arms race between *E. necator* and its grapevine host (9).

Next to gene duplications, CNVs within a species population can significantly affect its fitness (118). It has been shown, for example, that an increase in *CYP51* (*ERG11*) copy numbers, the gene encoding a key enzyme for ergosterol biosynthesis, creates a gene dosage effect that reduces the sensitivity of *E. necator* to demethylase inhibitor fungicides (7). In *B. graminis* f.sp. *hordei* (73, 119) and *B. graminis* f.sp. *tritici* (9), high levels of CNV in genes encoding CSEPs are thought to be major drivers of virulence and rapid adaptation to host genotypes. Our CNV analysis revealed that the CE-encoding gene *HI914_00624* exhibited the most dynamic changes in copy numbers, suggesting that it is a target of natural selection. Moreover, we found that *HI914_00624* is a member of a novel family of CE-encoding genes with multiple duplications in PM species. CEs are a large superfamily of structurally diverse, multifunctional enzymes that hydrolyze carboxylesters in natural and synthetic molecules (83), including pharmaceutical drugs, pesticides, environmental pollutants, and toxins. Due to their catalytic flexibility, they may have crucial roles not only in detoxifying cells from harmful compounds and metabolites but also in physiological processes such as lipid metabolism and energy homeostasis (120). We speculate that the putative CE encoded by *HI914_00624*, and its homologs in PM species, represents a new family of non-catalytic CEs, as they were poorly conserved in non-PM fungi and lack the conserved Ser-Asp/Glu-His amino-acid triad required for their proper function (83). Catalytic competence is thought to be the ancestral state of CEs, but several non-catalytic clades that have acquired new functions are present in higher eukaryotes (83, 121). Among fungi, *vdtD* from the opportunistic human pathogen *Paecilomyces variotii* encodes a putative non-catalytic CE that is part of a gene cluster mediating the biosynthesis of the antibacterial viriditoxin (122). It has been suggested that instead of acting as a hydrolase, vdtD could bind to the compound to protect the methyl ester from being hydrolyzed by endogenous hydrolases (123). These examples highlight the capability of CEs to evolve new functions, and it is, therefore, possible that the putative CE encoded by *HI914_00624* and its homologs in PMs has evolved new functions compared with ancestral CEs.

### Supplementary materials

All supplementary materials are available through Zenodo at https://doi.org/10.5281/zenodo.7738565 and include Supplementary Results, Supplementary Materials and Methods, Fig. S1 to S21, and Supplementary Tables S1 to S26.

## Data Availability

Raw sequencing reads generated in this study were deposited at NCBI SRA under accessions SRR18712274 through SRR18712279  (BioProject PRJNA627990 ). The annotated genome of EnFRAME01 was deposited at NCBI under accession JABETL000000000.1.
